# Statement of Retraction: Long non-coding RNA MEG3 attends to morphine-mediated autophagy of HT22 cells through modulating ERK pathway

**DOI:** 10.1080/13880209.2026.2719337

**Published:** 2026-08-24

We, the Editors and Publisher of *Pharmaceutical Biology*, have retracted the following article:

Gao, S., Li, E., & Gao, H. (2019). Long non-coding RNA MEG3 attends to morphine-mediated ­autophagy of HT22 cells through modulating ERK pathway. *Pharmaceutical Biology*, *57*(1), 536–542. https://doi.org/10.1080/13880209.2019.1651343

Following publication, concerns were raised by a third party regarding the integrity of the western blot images in the article. When approached for an explanation, the authors did not respond to our queries.

As verifying the validity of published work is core to the integrity of the scholarly record, we are therefore retracting the article. The corresponding author has been informed.

We have been informed in our decision-making by our editorial policies and the COPE guidelines.

The retracted article will remain online to maintain the scholarly record, but it will be digitally watermarked on each page as “Retracted”.

